# Quantum Biological Channel Modeling and Capacity Calculation

**DOI:** 10.3390/life2040377

**Published:** 2012-12-10

**Authors:** Ivan B. Djordjevic

**Affiliations:** Department of Electrical and Computer Engineering, College of Engineering, University of Arizona, 1230 E. Speedway Blvd., Tucson 85721, AZ, USA; E-Mail: ivan@email.arizona.edu; Tel.: +1-520-626-5119; Fax: +1-520-626-3144

**Keywords:** Quantum biology, bioinformatics, DNA quantum information, biological channels, channel capacity

## Abstract

Quantum mechanics has an important role in photosynthesis, magnetoreception, and evolution. There were many attempts in an effort to explain the structure of genetic code and transfer of information from DNA to protein by using the concepts of quantum mechanics. The existing biological quantum channel models are not sufficiently general to incorporate all relevant contributions responsible for imperfect protein synthesis. Moreover, the problem of determination of quantum biological channel capacity is still an open problem. To solve these problems, we construct the operator-sum representation of biological channel based on codon basekets (basis vectors), and determine the quantum channel model suitable for study of the quantum biological channel capacity and beyond. The transcription process, DNA point mutations, insertions, deletions, and translation are interpreted as the quantum noise processes. The various types of quantum errors are classified into several broad categories: (i) storage errors that occur in DNA itself as it represents an imperfect storage of genetic information, (ii) replication errors introduced during DNA replication process, (iii) transcription errors introduced during DNA to mRNA transcription, and (iv) translation errors introduced during the translation process. By using this model, we determine the biological quantum channel capacity and compare it against corresponding classical biological channel capacity. We demonstrate that the quantum biological channel capacity is higher than the classical one, for a coherent quantum channel model, suggesting that quantum effects have an important role in biological systems. The proposed model is of crucial importance towards future study of quantum DNA error correction, developing quantum mechanical model of aging, developing the quantum mechanical models for tumors/cancer, and study of intracellular dynamics in general.

## 1. Introduction

Recently, quantum biological studies have gained momentum, which can be judged by the number of recent publications related to this topic [1,2,3,4,5,6,7,8,9,10,11,12,13,14,15,16,17,18,19,20,21]. It has become evident that quantum mechanics has an important role in photosynthesis [8], magnetoreception [9], and evolution [5]. In photosynthesis, the Fenna–Matthews–Olson (FMO) complex supports the coherent transport of electron excitation over a short period of time, upon the photon absorption, to the reaction center where the energy gets converted into sugar. In the second phase of this interaction, the environment decoheres the system, which speeds up the excitation transfer by avoiding the system being trapped into the dark states. Further, the DNA replication and protein synthesis have been described by using Grover’s search algorithm [15]. Additionally, the quantum mechanical formalism for biological evolution has been established in [21].

There were also many attempts in an effort to explain the structure of genetic code and transfer of information from DNA to protein by using the concepts of quantum mechanics [3,5,7]. For instance, the general quantum mechanical model to describe the transfer of information from DNA to protein by using the quantum information theory is proposed by Karafyllidis [3]. However, given the high complexity of the problem [22], the determination of *quantum biological channel capacity* is still an open problem. On the other hand, the classical biological channel capacity has been determined by Yockey [10]. Yockey developed a discrete memoryless classical biological channel model, and explicitly derived the transitional probabilities among amino acids. Namely, he represented the information transfer from DNA to protein as a communication problem and determined the corresponding classical biological channel capacity by maximizing the mutual information between DNA and protein. Interestingly enough, there are certain papers that question the role of quantum information in biological processes [12]. The main justification is that the entanglement in living system is limited to a very short time and small regions, and the decoherence is too high for quantum entanglement to be relevant on the molecular level in the ambient conditions. However, in photosynthesis, as indicated above, this decoherence phase is essential to avoid the system being caught in dark states. Moreover, it is possible to represent the genetic coding using the quantum error correction concepts. Even though that decoherence is going to introduce errors, these errors can be corrected by the “quantum decoder,” performing the mRNA to protein translation. Another argument, also known as *k*_B_*T*-argument, claims that whenever interaction energies are smaller than *k*_B_*T* at room temperature, the corresponding quantum effect cannot persist. However, the electron clouds of complementary DNA strands experience dipole–dipole interaction, resulting in attractive van der Waals bonding [14], with interaction frequency *ω* being in the optical range, suggesting that 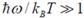
Therefore, since the electronic system of DNA is globally in the ground state, the DNA system must be locally in a mixed state. Given the uncertainty principle, it is impossible to distinguish whether a local mixed state is a result of temperature or entanglement. 

In this paper, we develop the quantum biological channel model suitable for study of quantum information transfer from DNA to proteins, as well as the quantum DNA error correction. The quantum channel model is derived based on codon transition probabilities. We assume that the quantum genetic noise is introduced by the imperfect transcription process, DNA point mutations, insertions, deletions, and imperfect translation process. By using the Holevo–Schumacher–Westmoreland (HSW) theorem [11], we adopt the proposed model to determine biological quantum channel capacity. We observe several cases of interest: (i) completely mixed state approach, in which the different codon basekets (basis vectors) representing the same amino acid occur with the same probability; (ii) by representing an amino acid as a superposition of eigenvectors spanning the subspace of DNA Hilbert space, and, (iii) by selecting an eigenvector representing an amino acid from subspace at random. We will show later in the paper that the quantum biological channel capacity is higher than classical channel capacity, suggesting that the quantum mechanics has an important role in biological systems. 

## 2. Quantum Biological Channel Model Suitable for Study of Quantum Information Transfer from DNA to Proteins

**Figure 1:**
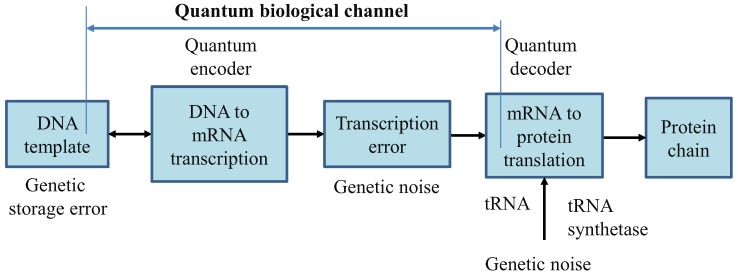
Generic quantum channel model describing the information flow from DNA to protein. It is assumed that quantum genetic noise introduces the random errors. In this model, the classical information, representing the information for a single polypeptide synthesis, is transmitted over the quantum genetic channel. DNA template is also a subject of mutations and, as such, is also represented as a part of biological quantum channel.

In Figure 1, we describe the generic quantum biological model suitable to study the flow of information from DNA to protein. The process of transcription is interpreted as encoding, the various errors introduced during replication, transcription or translation are considered as a source of genetic noise, while the process of translation is interpreted as decoding. Notice that this interpretation is consistent to the classical model defined by Yockey [10]. The process of translation is also prone to errors, which can also be contributed to the genetic noise. Therefore, all errors introduced in any stage shown in Figure 1 are called “genetic noise.” The transcription, DNA point mutations, insertions, deletions, and translation can all be interpreted as the quantum channel. It is assumed that genetic noise introduces random errors, which can be classified into several broad categories. The first types of errors, “storage errors,” occur in DNA itself as it is an imperfect storage of genetic information. The second types of errors are introduced during the DNA replication process. (For instance, the probability of error during replication is ~10^−7^ per base pair [15].) The third types of errors, transcription errors, are introduced during DNA to mRNA transcription process. The fourth type of errors, translation errors, is introduced during the translation process. 

Therefore, the key difference with respect to communication systems, where both encoders and decoders are implemented from perfect gates, in biological systems DNA replication, DNA to mRNA transcription, and mRNA to protein translations, are imperfect processes. This difference requires redefining the channel model, as it is done in Figure 1.

We assume that the problem with the burst of errors, introduced for example by an alpha particle passing through a segment of DNA, have been resolved by duplicate genes. Moreover, the introns may serve as interleavers in eukaryotic genes, suggesting that quantum errors indeed appear in a random fashion. The genome, which represents the ensemble of genetic codewords, is pre-recorded in DNA sequence by using the nucleotide alphabet composed of four symbols: Adenine (A), guanine (G), cytosine (C) and thymine (T). The fourth base is replaced in RNA by uracil (U). The DNA codewords contain the information for protein synthesis. The mRNA consists of three-symbol words known as codons. Multiple codons can correspond to the same amino acid.

The point mutations are caused by tautomeric forms of nucleic acids [13,16]. There exist two main forms of tautomerism: (i) *amino-imino* tautomerism given by [13]

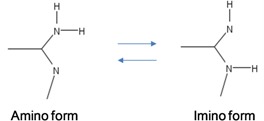

and *keto-enol* tautomerism given by [13]

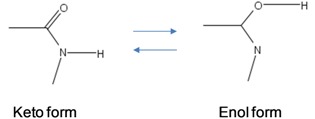


**Figure 2:**
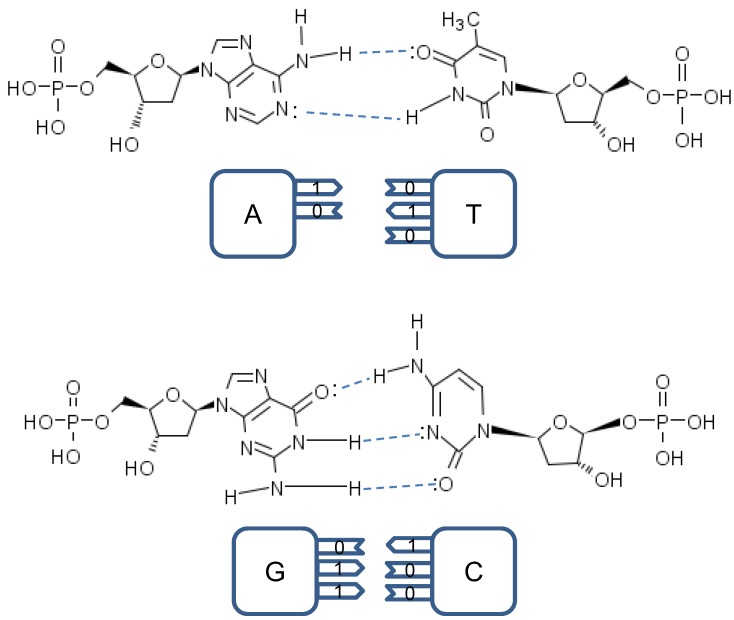
Standard nucleic acids’ pairing together with schematic representation. In schematic representation the symbol 1 is used to denote the “donor” of a proton, while symbol 0 to denote “acceptor” of a proton.

**Figure 3:**
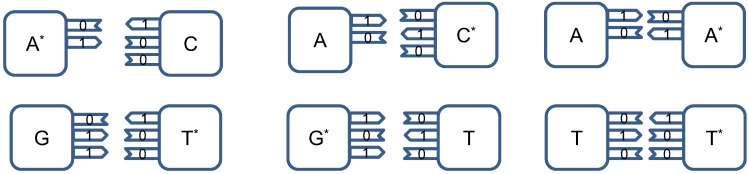
The tautomeric nucleic acids’ pairing.

**Figure 4:**
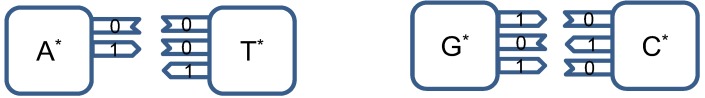
Tautomeric forms are responsible for random errors introduced within DNA, when DNA is used for long-term storage of genetic information.

The amino and keto forms are considered as standard forms. Namely, the pairing of nucleotides is specific to 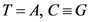
combinations, with 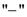
representing the hydrogen bond (H-bond), as shown in Figure 2. In the same figure, we provide the schematic representation in which the “donors” of protons are denoted with symbol 1, while the “acceptors” of protons are denoted with symbol 0. However, in tautomeric forms A^*^, G^*^, C^*^ and T^*^_,_ a proton involved in H-bond has been moved from one electron lone pair to another, which is illustrated in Figure 3. During either DNA replication or translation, the tautomeric nucleic acids bind with non-complementary nucleic acids introducing the mutations. For instance, tautomeric A^*^ binds with C instead of T (U in mRNA), tautomeric C^*^ binds with A, G^*^ binds with T (U) and T^*^ (U^*^) with G. It is also possible that both bases in DNA pair to be in the tautomeric forms as illustrated in Figure 4. Namely, when DNA is used for long-term storage of genetic information, the proton tunneling (tautomeric forms’ creation) can contribute to random errors introduction. The probability of occurrence of tautomeric nucleic acids’ forms is low so that it makes sense to assume that errors introduced by tautomeric forms are random. However, the presence of mutagens, carcinogens, electromagnetic radiation and/or proton bombardment can either increase the probability of occurrence of tautomeric forms or damage the bases, and consequently introduce the storage errors. In addition to increasing the rate of spontaneous mutations, the mutagens can cause the induced mutations due to deamination, oxidation, and alkylation. The DNA is the subject of continuous mutations and damages, and the cell has various mechanisms to deal with damages, including direct reversal (such as photoreactivation) mechanism, various damaged bases excising mechanisms (nucleotide excision repair-NER, base excision repair-BER and nucleotide mismatched repair-NMR), single-strand damage repair, double-strand breaks repair, and translesion synthesis (TLS). In this paper, we assume that only the point induced mutations, transitions or transversions, have remained upon the DNA repair process. 

Even though the probability of occurrence of tautomeric forms in normal ambient conditions is low, we can associate corresponding probability amplitude between normal and tautomeric forms, and represent the nucleic acid states as quantum states. For instance, the superposition states corresponding to T and C can be represented as:

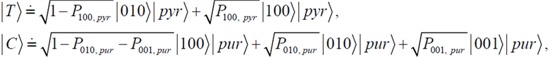

where with 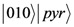
we denoted the standard form of T, while 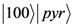
the corresponding tautomeric form T^*^ that occurs with probability *P*_100, pyr_ (10^−5^–10^−3^). We use the notation 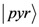
(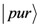) to denote that corresponding base is of pyrimidine (purine) type so that we can uniquely distinguished among different tautomeric forms. Strictly speaking, the probability amplitudes are complex numbers; however, from a quantum information theory point of view, the representation given above is sufficient.

To determine the exact probability of the occurrence of the tautomeric form, we can use the *double-well* model and tunneling effect in a fashion similar to that first described in [13]. By close inspection of Figure 2, we can see that H-bonds in standard forms are asymmetric. Further, the protons have two equilibrium positions, for instance:

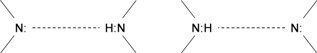


**Figure 5:**
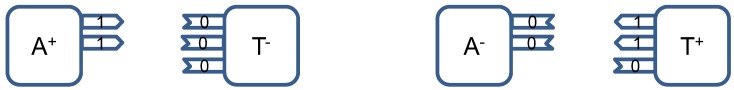
The illustration of nonstandard splitting during the replication process. The superscript + is used to denote the existence of additional proton, while the superscript – is used to denote a missing proton.

**Figure 6:**
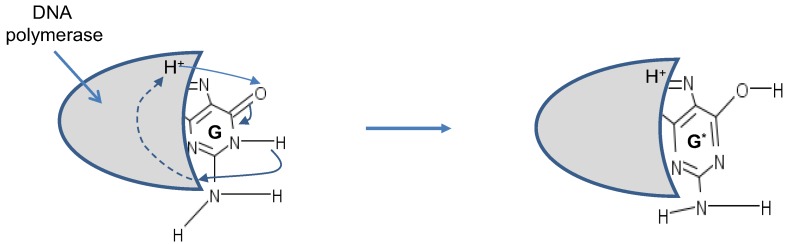
Tautomeric formation of G^*^ by proton transfer between DNA polymerase (active region shown by shaded area) and G.

The principles of quantum mechanics indicate that the proton can move between these two equilibrium positions, so that it makes sense to observe the corresponding quantum states as the superposition of these two equilibrium states, with probability amplitude not necessarily 1/√2 (the exact probability amplitude needs to be determined either experimentally or by using double-well model [13]). The hydrogen bonds are weak bonds; and as it has been shown in [14], the electron clouds of complementary DNA strands experience dipole–dipole interaction, resulting in attractive van der Waals bonding. Therefore, the superposition of basekets can be caused by either van der Waals interaction or thermal fluctuations. When the DNA polymerase reads out the genetic information, it separates two DNA strands, and the nonstandard proton position can cause the error. For instance, the separation of AT in Figure 2 can result into a nonstandard splitting as shown in Figure 5. The nonstandard splitting, illustrated in Figure 5, is responsible for time-shift deletion errors introduced during DNA replication. A nucleotide deletion error causes the amino acids inserted after the deleted nucleotide to be incorrect. The process of recognition of DNA polymerase and corresponding nucleotide can also be responsible for creation of tautomeric forms, which is illustrated in Figure 6.

The mutation rates can vary even within the same genome of a single organism, with typical values 10^−9^–10^−8^, particularly in bacteria. (Interestingly enough, the error-prone polymerase genes in human have the mutation rates 10^−3^–10^−2^, as shown by Johnson [17].) In rapidly reproducing bacteria, even the mutation rates of the order 10^−9^ accumulate fast over the time, leading to the resistance of bacteria to antibiotics [18]. Therefore, even though the tautomeric forms occur with low probability (10^−5^–10^−3^), they introduce the occasional DNA storage and replication errors, which are responsible for mutations, aging and evolution. 

The models described above will be used to determine the probability of single and double codon errors (the probability of triple codon errors is so small that can be considered negligible). Notice that here we are concerned with small-scale mutations; such as point mutations, deletions, and insertions; rather than large-scale mutations (amplifications, chromosomal region deletions, chromosomal translocations, heterozygosity loss, *etc.*). Therefore, it is of crucial importance to determine how the quantum channel capacity changes with respect to single base error probability *p*. (Notice that double-base error probability is proportional to *p*^2^ and can be neglected when the probability *p* is low.) It is important to point out that some of the errors introduced during the DNA replication are corrected by various DNA repair processes. Repair enzymes are able to recognize improperly paired nucleotides, remove out the wrong nucleotides and replacing them with the correct ones. Unfortunately, some of the replication errors stay uncorrected during DNA repair and thus represent permanent mutations. We assume that these permanent mutations contribute to the base error probability *p*. Given the fact that three bases determine the codon, representing the amino acid in protein synthesis, the single base error does not necessarily result in amino acid error. Since the genetic information is encoded in codons, it makes sense to assume that each codon represents a baseket. Because there exist 64 codons, the corresponding Hilbert code space is 64-dimensional. Similarly to the model due to Karafyllidis [3], we assume that each codon is a baseket in this Hilbert space. For instance, the basekets (codons) corresponding to Ile are |AUU〉, |AUA〉, and |AUC〉, and they span the subspace of Hilbert code space. In what follows, we describe three interesting scenarios. In scenario (i), we assume that |Ile〉-state is a completely mixed state, a statistical mixture of basekets each occurring with the same probability. The corresponding density state (operator) of an amino acid is given by 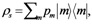
where *p_m_* describes the probability that a member of the ensemble (codon baseket) has been prepared in state |*m*〉. For instance, *m*⋴{AUU, AUA, AUC} for Ile, and *m*⋴{UUU, UUC} for Phe. In scenario (ii), we assume that |Ile〉-state is a superposition of eignekets of corresponding Hamiltonian [3]:

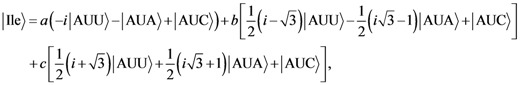

where *a*, *b* and *c* are probability amplitudes. (In the lack of experimental data, we assume that 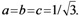) In similar fashion, the |Phe〉-state is a superposition of eigenkets (eigenvectors) |UUC〉+|UUU〉 and |UUC〉-|UUU〉:

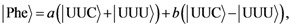

where *a* and *b* are corresponding probability amplitudes. In case (iii), we select one of the eigenkets from equations above at random. The same strategy is applied to other amino acids. 

**Figure 7:**
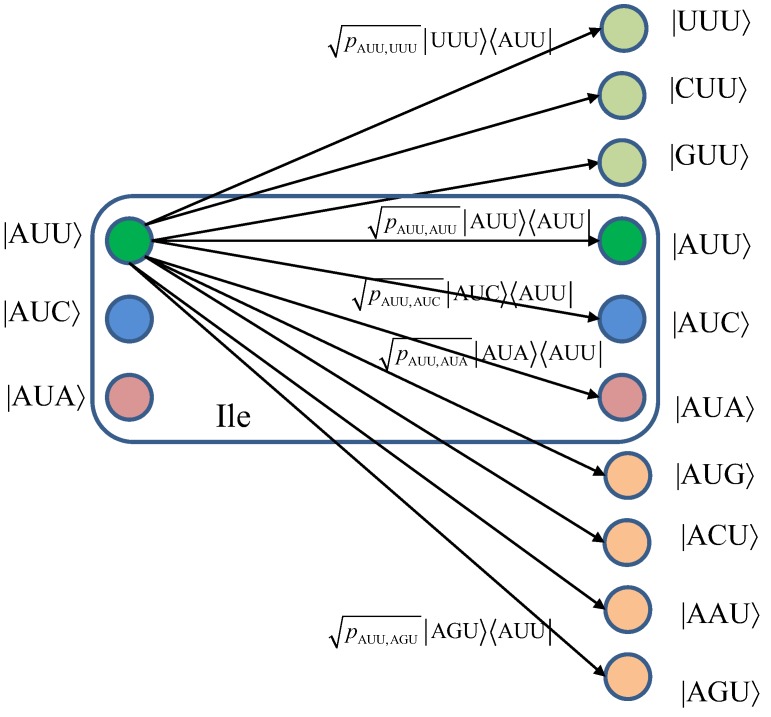
The quantum biological channel transition diagram for basekets corresponding to Ile. Only transitions from baseket |AUU〉 due to the errors in single base of codon are shown. The *p_m_*_,*n*_ denotes the transition probability from baseket |*m*〉 to baseket |*n*〉, where *m*⋴{AUU,AUC,AUA} and *n* could be any of 64 basekets. The Kraus operator *E_m_*_,*n*_ is obtained as 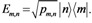.

**Figure 8:**
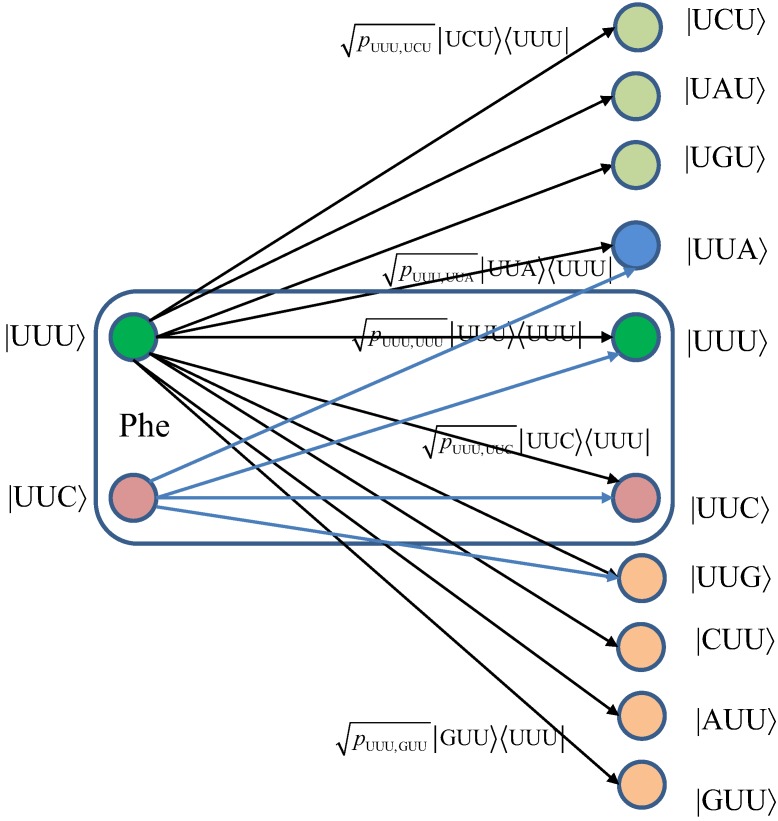
The quantum biological channel transition diagram for basekets corresponding to Phe.

We turn our attention to the quantum channel model, which is applicable to all three scenarios discussed above. When the baseket |*m*〉, representing one of codons (*m*), is transmitted over the quantum biological channel, it can be detected on the receiver (protein) side as baseket |*n*〉 (*n* ≠ *m*) due to the presence of genetic noise. To completely characterize this quantum channel model it is essential to determine baseket transition probabilities. As an illustration, in Figure 7, we provide the transition diagram corresponding to Ile, while in Figure 8, we provide the transition diagram corresponding to Phe. For Ile, only transitions caused by single base error in baseket |AUU〉 are shown. On the other hand, for Phe the single base error caused transitions for |UUU〉 have been shown. Other transitions can easily be obtained by following the same strategy. With *p_m_*_,*n*_ we denoted the transition probability from baseket |*m*〉, representing the initial codon, to baseket |*n*〉, representing the final codon; while corresponding Kraus operator is given by 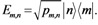
Notice that certain codon base errors will not result in a different amino acid, as illustrated in Figure 7 and Figure 8. As a remark, the model described in Figure 7 and Figure 8 is used to describe the collective action of various types of errors (DNA storage errors, DNA replication errors, transcription errors and translation errors) on protein synthesis and the amount of DNA information transfer from DNA to protein.

True transitional probabilities can be obtained either from experimental data or by employing Löwdin’s double-well model, while for illustrative purposes we will assume that single base errors are dominant and independent of each other. The model described above is applicable to both spontaneous mutations and induced mutations (caused by mutagens, be they of chemical origin or introduced by radiation). In induced mutations, the base error probability increases as the concentration of corresponding chemical (NH_2_OH, BrdU, *N*-ethyl-*N*-nitrosourea, Ochratoxin A, EtBr, *etc.*) or the radiation level increases. Once the level of induced mutations goes well above the natural level, the quantum channel capacity starts to decrease dramatically as shown later in Section 4.

## 3. Quantum Biological Channel Capacity

In quantum information theory, developed by Schumacher (see [11] and references therein), a quantum channel can be described by the transformation of an input density matrix *ρ_s_* to the output density matrix *ρ_s_’*. This transformation, described by the quantum operation (superoperator) *U*, can be represented as the following mapping 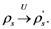
Clearly, the super-operator *U* cannot be unitary due to the decoherence effects. However, the total evolution operator of quantum system and environment (ambient) can be represented by unitary operator *U_s_*_,*E*_. Without loss of generality, let us assume that the environment (ambient) *E* is initially in a pure state |0*_E_*〉. Hence, the expression for super-operator under this initial condition can be written as Equation 1.

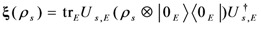
(1)
where the partial trace tr*_E_*(·) is taken with respect to the environmental degrees of freedom. Consider equation (1) as a completely positive linear transformation acting on the density matrix, which can be reconstructed as Equation 2.

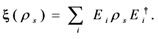
(2)

The corresponding Kraus operators *E_i_* in (2) must satisfy the completeness relationship such that

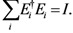


For the quantum biological channel, the Kraus operator *E_m_*_, *n*_ performing the transformation from codon baseket |*m*〉 to |*n*〉 can be constructed according to the transition probabilities *p_m_*_,*n*_, as discussed earlier. Clearly, the generic quantum biological channel model consists of 4,096 Kraus operators. An arbitrary state, |*ψ*_in_〉= ∑*_m_* *α_m_*|*m*〉, represents the amino acid in genome; after transcription, point mutations, translation, and other genetic noise sources can be represented as Equation 3.

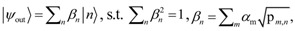
(3)
while the resulting density matrix can be expressed as Equation 4.

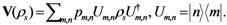
(4)

We can rewrite the previous equation in terms of Kraus operators 
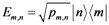
, as Equation 5.

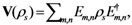
(5)
which is consistent with equation (2), after replacing double-indexing with single-indexing. Notice that partial trace over the environment qubit has been omitted due to the fact that a mixing process can be described either with or without a fictitious environment [11]. In other words, 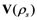
is equivalent to the density operator 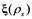
after tracing out the environment. 

The genetic noise introduces uncertainty about the genome, and the amount of information related to initial density state *ρ_s_*, which can be expressed as [11] (Equation 6).

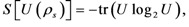
(6)
where *S*[(*U*(*ρ_s_*)] denotes the von Neumann entropy. The elements in the *i*-th row and the *j*-th column of the information matrix *U* can be found as Equation 7.

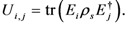
(7)

Each element of *U* measures the exchangeable amount of information between the system and the environment during its interaction. With initial environment states being the pure states, *S*(*U*) gives the entropy of the environment. By employing the HSW theorem [11], the quantum channel capacity can be calculated as Equation 8.

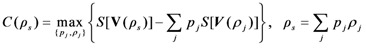
(8)
where the maximization is performed over all ensembles {*p_j_*, *ρ_j_*} of possible input states *ρ_j_*. By choosing *a priori* probabilities of codons as *p_i_*= 1/61 and *p_i_*=0 for stop codons (UAA, UAG, and UGA), the maximum information rate can be achieved since the source is discrete.

## 4. Results

**Figure 9:**
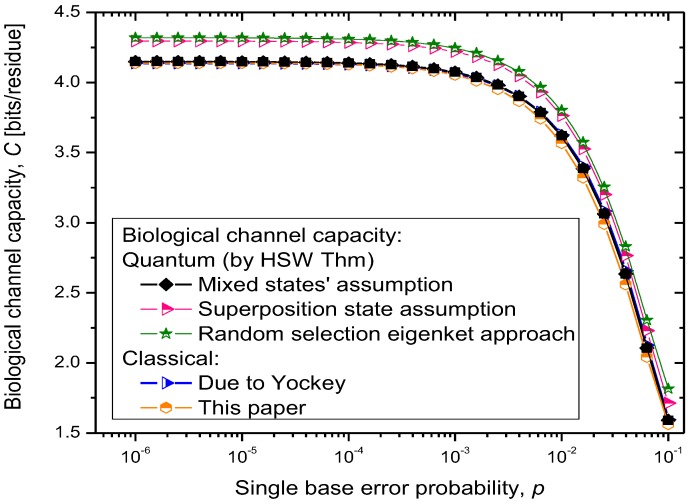
Quantum biological channel capacity against the single base error probability.

The results of quantum channel capacity with respect to the single base error probability are depicted in Figure 9. The results of calculations are obtained by using the Equations 6–8. 

Clearly, for the scenario (i), the quantum channel capacity is very similar to that of the classical channel, which is expected as the quantum biological information is represented as *statistical* (classical) mixture of codon basekets. This case can be considered as a lower bound on quantum biological channel capacity. The use of superposition state assumption, scenario (ii), results in higher quantum channel capacity, as shown in Figure 9. Finally, the random selection of Hamiltonian eigenkets, spanning the subspace of corresponding amino acid, results in even higher quantum channel capacity, which can be interpreted as an upper bound on the quantum channel capacity. Therefore, the numerical results shown in Figure 9 suggest that quantum effects could play a role in increasing robustness of information processing in biological systems. Even though the coherent quantum channel model shows small improvements in biological channel capacity than classical channel models, in addition to improved reliability of biological information processing, the quantum coherence could also improve the DNA replication rates as suggested by Patel [15]. Namely, as it has been advocated by Patel, the enzymes could provide the shielded environment for quantum coherence to be preserved sufficiently long for the completion of the base-paring process. When the base error probability is larger than 10^−2^, the quantum biological channel capacity decreases dramatically, as shown in Figure 9, and in that regime the quantum coherence of biological systems cannot be preserved. We conclude that for the base error probability higher than 10^−2^ the classical biological processes might have the same role as quantum processes as the gap between quantum and classical models becomes negligible. For base error probabilities close to 0.1, the biological channel capacity drops dramatically, suggesting that the cell is no longer capable of performing protein synthesis. Notice that in this analysis, different contributors to genetic noise (storage, replication and translation errors) are included in the base error probability *p*. 

**Figure 10:**
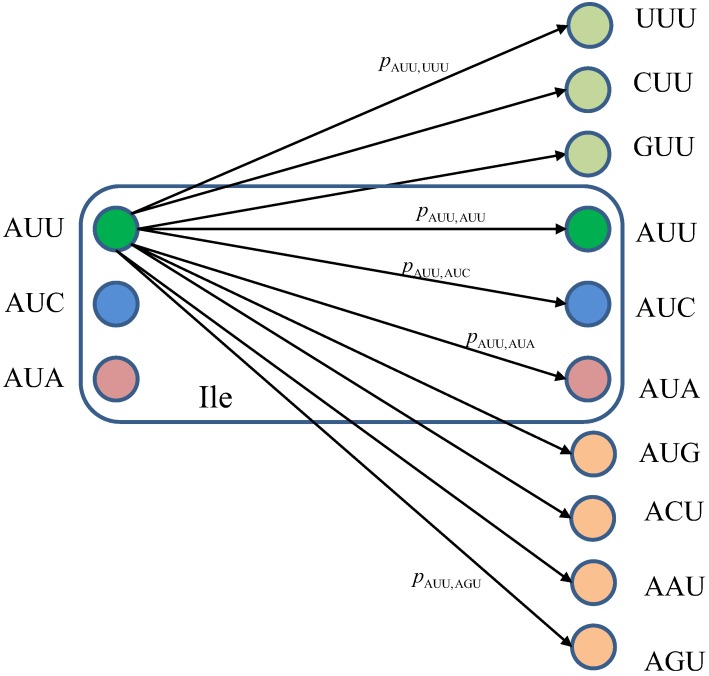
The classical biological channel transition diagram for codons corresponding to Ile. The *p_m_*_,*n*_ denotes the transition probability from codon *m* to codon *n*, where *m*⋴{AUU,AUC,AUA} and *n* could be any of 64 codons.

For completeness of presentation, in Figure 9, we also report the results of classical biological channel capacity obtained by using the model credited to Yockey [10], which are in excellent agreement with classical channel capacity calculations obtained by developing the equivalent classical model to that shown in Figure 7 and Figure 8 (by simply converting codon basekets to classical nonbinary symbols), which is illustrated in Figure 10. The *p_m_*_,*n*_ denotes the transition probability from codon *m* to codon *n*, *p_mn_*=*P*(*n*|*m*), where *m*⋴{AUU,AUC,AUA} and *n* could be any of 64 codons. The codon transition probabilities are employed to evaluate the classical channel capacity, defined as Equation 9.

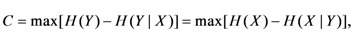
(9)
where *H*(*Y*) [*H*(*X*)] stands for the biological channel output (input) entropy, and *H*(*Y*|*X*) [*H*(*X*|*Y*)] represents the conditional entropy (or the equivocation) of biological channel output (input) given the biological channel input (output) *X* (*Y*). The entropy of biological channel output and conditional entropy are defined respectively as Equations 10 and 11.

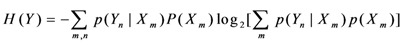
(10)

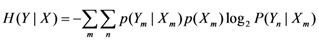
(11)
where {*p*(*X_i_*)} denotes the probability of occurrence of codons in DNA, and {*p*(*Y_j_*|*X_i_*)} denotes the conditional probability of the received codons {*Y_i_*} given the transmitted codons {*X*_i_}. Since the source information encrypted in DNA is discrete, the maximum information rate in (9) is achieved for the uniform distribution of codons; that is *p_i_*=0 for stop codons (UAA, UAG, and UGA) and *p_i_* = 1/61 for other codons. The classical biological channel capacity curve in Figure 9 (the orange curve) is obtained by using Equations 9–11 and model from Figure 10, and represents a lower bound on biological channel capacity. The transition probabilities *p_mn_* required in Equations 10 and 11 are obtained numerically by using the model shown in Figure 10 and the theory of Markov chains. These transition probabilities are also used in the quantum biological channel model, see Figure 7 and Figure 8, to determine probability amplitudes needed for calculation of quantum channel capacity by HSW theorem (by using Equation 8).

## 5. Conclusions

We presented the quantum biological channel model suitable for study of quantum information transfer from DNA to proteins. By using the transition probabilities of codon basekets, the quantum channel model is developed by exploiting the operator-sum representation. The quantum genetic noise originates from the imperfections in transcription process, DNA point mutations, insertions, deletions, and imperfect translation process. This quantum channel model has been adopted to evaluate both quantum and classical channel capacities in the presence of genetic noise. The proposed quantum biological channel model is of crucial importance for future study towards quantum DNA error correction and study of various biological systems from a quantum information theory point of view. It can also be used to develop the quantum mechanical model of aging, and the quantum mechanical models for tumors and cancer. The similar channel model can be developed to study other aspects of intracellular dynamics. 

Regarding the complexity, the quantum processes in the cell do not require very complicated quantum information-processing devices. For instance, the DNA replication process described in terms of Grover’s search algorithm requires only the rotation and oracle operators, which can be quite straightforwardly implemented [15]. Based on discussion above, it is clear that quantum information theory can indeed be used to describe various biological processes in the cell. 
